# Disease onset and aging in the world of circular RNAs

**DOI:** 10.15761/jts.1000158

**Published:** 2016-08-31

**Authors:** Kenneth Maiese

**Affiliations:** Cellular and Molecular Signaling, Newark, New Jersey 07101, USA

**Keywords:** aging, apoptosis, autophagy, β-catenin, biomarkers, cancer, cardiovascular disease, cyclin-dependent kinase 2 (CDK2), cyclin-dependent kinase inhibitor 1 (p21), cell cycle, circular RNA, diabetes mellitus, endothelial cells, forkhead transcription factors, FoxO, metabolism, microRNA, p21, programmed cell death, reactive oxygen species, senescence, stem cells, transcription factors, vascular smooth muscle cells, Wnt signaling

## Abstract

Circular ribonucleic acids (circRNAs) are non-coding RNAs of approximately 100 nucleotides in length with thousands of members in mammalian cells. The presence of circRNAs is believed to be even greater than that of messenger RNAs. Identification of circRNAs occurred approximately 37 years ago with the subsequent demonstration that covalent bonds are necessary for the unique circular structure of these ribonucleic acids. However, present understanding of the complex biological role of circRNAs remains limited and requires further elucidation. CircRNAs may impact aging, multiple disorders, function as biomarkers, and are able to regulate gene expression by acting as effective microRNA (miRNA) sponges. New work suggests that circRNAs are vital for the modulation of cellular senescence and programmed cell death pathways such as apoptosis. These non-coding RNAs can control cell cycle progression, cellular proliferation, and cellular survival impacting disorders linked to aging, cardiovascular disease, and atherosclerosis through pathways that involve cyclin-dependent kinase 2 (CDK2), cyclin-dependent kinase inhibitor 1 (p21), and mammalian forkhead transcription factors. In addition, circRNAs can oversee cellular metabolism and disorders such as diabetes mellitus through the regulation of insulin signaling as well as limit tumor progression through Wnt signaling and β-catenin pathways. Further understanding of the biology of circRNAs offers great promise for the targeting of novel strategies against a wide spectrum of disease entities.

## Circular RNAs, cellular senescence, and programmed cell death

Circular ribonucleic acids (circRNAs) are non-coding RNAs of approximately 100 nucleotides in length that were initially identified as being circular in nature [1,2]. Subsequently, these non-coding RNAs were later demonstrated to have covalent bonds that maintain the circular structure. In the body, circRNAs have thousands of members present in mammalian cells. In eukaryotic cells, circRNAs can be composed of the loop portion of intronic lariats, plant viroids, intermediates of transfer RNAs (tRNAs), antisense transcripts, circRNAs from noncoding genes, and exonic RNAs [3]. Knowledge in regards to circRNAs is rapidly growing. It is now recognized that the isoform of circRNA has a greater expression than messenger RNA (mRNA) [4]. In addition, circRNAs have both *cis* and *trans* regulation. CircRNAs have been shown to regulate gene expression through the sponging of microRNAs (miRNAs) [5].

New work has highlighted the role of circRNAs in both cellular senescence and cellular survival. It has recently been demonstrated that circRNA generated from the mammalian forkhead transcription factor Foxo3 plays a role in cellular senescence and aging. Presently in the mammalian forkhead transcription factor family, more than 100 forkhead genes and 19 human subgroups that range from *FOXA* to *FOXS* exist [6–8]. In regards to mammalian FOXO proteins, this group is assigned to the O class of the forkhead box class transcription factors. The family consists of FOXO1, FOXO3, FOXO4, and FOXO6 [9]. FOXO proteins are expressed in all tissues of the body [10]. For FoxO3, this mammalian transcription factor may have an important role in erythroid cell growth [11], endothelial vascular cell survival [12,13], hippocampal neuronal injury [14,15], neuronal cortical disease [10,16,17], and behavior disorders [18]. In the cardiovascular system during aging, circRNA generated from Foxo3 (circ-Foxo3) is expressed in aged patients and murine experimental models. Silencing circ-Foxo3 blocks senescence in mouse embryonic fibroblasts and over-expression of circ-Foxo3 results in cell senescence [19]. In relation to the mechanisms that may account for the cellular senescence, circ-Foxo3 appears to block cell cycle progression by binding to the cell cycle proteins cyclin-dependent kinase 2 (CDK2) and cyclin-dependent kinase inhibitor 1 (p21) to prevent cellular proliferation [20]. Additional evidence exists for the link between circRNAs and the onset of aging processes. For example, with advanced age, increased expression of circRNAs has been demonstrated in the skeletal muscles of monkeys [21].

CircRNAs also oversee cellular survival through programmed cell death involving apoptosis [22,23]. In vascular smooth muscle cells and macrophages, circular antisense non-coding RNA in the INK4 locus (circANRIL) can prevent exonuclease-mediated pre-ribosomal RNA processing, ribosome biogenesis, and proliferation of cells that may lead to atherosclerosis through the induction of apoptosis [24]. It is conceivable that circANRIL could be protective against progressive cardiovascular disease. CircRNA also can function as an endogenous miR-223 sponge to inhibit cardiac hypertrophy and heart failure [25]. However, circRNAs may not always be protective against apoptotic pathways. During cell models of ischemia-reperfusion injury, up-regulation of specific circRNAs may foster apoptotic cell injury [26]. In experimental models of myocardial infarction, the circRNA Cdr1as could increase cardiac infarct size and function as a sponge for miR-7a, a protective agent in this model [27].

## Circular RNAs, metabolism, and cellular proliferation

Given the role of circRNAs in senescence, aging, and cell death, it is of interest to learn that circRNAs may control these processes through proliferative pathways that involve cellular metabolism and Wnt signaling [28]. During cellular metabolism, circRNAs may have a significant role in the development of diabetes mellitus (DM) [29]. DM affects the global population and is increasing in incidence throughout the world [30,31]. Approximately 350 million individuals currently have DM and an additional 8 million individuals are believed to be undiagnosed at present [30,32]. CircRNA Cdr1as may regulate insulin secretion through miR-7. Cdr1as is a sponge and inhibitor of miR-7. Without modulation of miR-7 expression, miR-7 can foster the progression of DM. Cdr1as appears to interact with miR-7, block its activity, and increase insulin content and secretion in islet cells [33].

CircRNAs also can control cellular growth through Wnt signaling pathways and function as biomarkers for disease progression and treatment Wnt proteins are cysteine-rich glycosylated proteins that oversee multiple cellular processes including neuronal development [34,35], musculoskeletal development [36,37], vascular growth [38], immunity [39], fibrosis [40,41], and stem cell proliferation [30,42,43]. However, Wnt signaling pathways also can lead to tumorigenesis since Wnt proteins are proliferative in nature [34,44–47]. CircRNAs have been reported to have a protective effect during colorectal cancer. cir-ITCH expression was found to be down-regulated in colorectal cancer when compared to normal surrounding tissue. Yet, cir-ITCH was found to be able to increase the level of ITCH that can inhibit the Wnt/β-catenin pathway and block colorectal tumor progression [48]. In regards to biomarker disease assessment, circRNAs may offer the ability to track disease progression such as during hepatocellular carcinoma [49]. Yet, oncology is not the only discipline that circRNAs may function as relevant biomarkers. For example, in patients with psychiatric disease, circRNAs may be both a diagnostic and therapeutic biomarker for major depressive disorder [50].

## Future considerations

As non-coding RNAs, circRNAs are ubiquitous, have thousands of members, and can regulate gene expression by functioning as effective miRNA sponges. Since circRNAs are present in exosomes, these non-coding RNAs have the ability to impact multiple cellular responses throughout the body. Under several conditions, circRNAs may control disease progression and are considered important biomarkers for multiple disorders. CircRNAs appear to be critical for the control of cellular senescence and cellular death pathways such as apoptosis. CircRNAs interface with multiple pathways that include cyclin-dependent kinase 2 (CDK2), cyclin-dependent kinase inhibitor 1 (p21), mammalian forkhead transcription factors, insulin signaling, Wnt, and β-catenin pathways. Ultimately, circRNAs may have control over aging dependent pathways, cell survival during acute injury, metabolic homeostasis, and tumorigenesis. Given that the identity of circRNAs occurred approximately 37 years ago [2], we currently have only a small grasp of the role that circRNAs play in disease onset and aging. Further efforts are clearly warranted to fully elucidate the biology of these unique non-coding RNAs in the body.
